# A New Miocene-Divergent Lineage of Old World Racer Snake from India

**DOI:** 10.1371/journal.pone.0148380

**Published:** 2016-03-02

**Authors:** Zeeshan A. Mirza, Raju Vyas, Harshil Patel, Jaydeep Maheta, Rajesh V. Sanap

**Affiliations:** 1 National Centre for Biological Sciences, Tata Institute of Fundamental Research, Bangalore 560065, India; 2 505, Krishnadeep Towers, Mission Road, Fatehgunj, Vadodra 390002, Gujarat, India; 3 Department of Biosciences, Veer Narmad South Gujarat University, Surat-395007, Gujarat, India; 4 Shree cultural foundation, Ahmedabad 380004, Gujarat, India; State Natural History Museum, GERMANY

## Abstract

A distinctive early Miocene-divergent lineage of Old world racer snakes is described as a new genus and species based on three specimens collected from the western Indian state of Gujarat. ***Wallaceophis* gen. et. *gujaratenesis* sp. nov.** is a members of a clade of old world racers. The monotypic genus represents a distinct lineage among old world racers is recovered as a sister taxa to *Lytorhynchus* based on ~3047bp of combined nuclear (cmos) and mitochondrial molecular data (cytb, ND4, 12s, 16s). The snake is distinct morphologically in having a unique dorsal scale reduction formula not reported from any known colubrid snake genus. Uncorrected pairwise sequence divergence for nuclear gene cmos between ***Wallaceophis* gen. et. *gujaratenesis* sp. nov.** other members of the clade containing old world racers and whip snake is 21–36%.

## Introduction

Colubrid snakes are one of the most speciose among serpents with ~1806 species distributed across the world [1–5]. Colubridae has had a complex taxonomic history and many attempts to break down this family in smaller units have thus far failed [2,6,7] and only recent phylogenetic analysis by several authors have addressed and resolved some of the taxonomic flux [2,8–10]. Among colubrids, racer and allied snakes have had immensely unstable state of taxonomy (especially members referred to the genus *Coluber s*.*l*. Linnaeus) and only in recent years some of it has been resolved however most remain unattended, especially Asian taxa (see [7,11–17].

**Fig 1:**
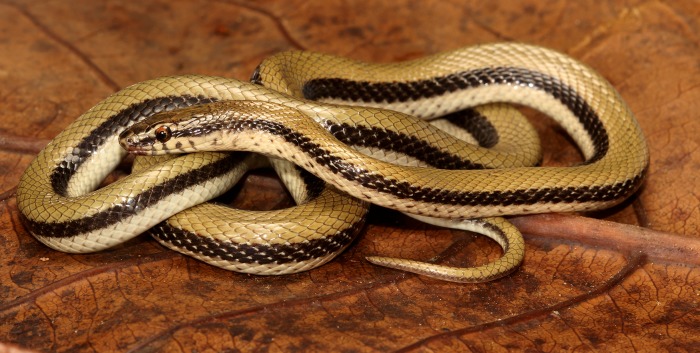
*Wallaceophis* gen. et. *gujaratensis* sp. nov. holotype male NCBS HA-105 in life. Photo by Zeeshan Mirza.

Vyas & Patel [18] depicted an uncollected snake in Fig 1 from Bhavnagar with two black longitudinal stripes to the genus *Coronella* while presenting new locality records for *C*. *brachyura* from Gujarat state. *Coronella brachyura* is a small sized snake lacking longitudinal stripes and a presubocular too [19] which raised doubts on the identity of the striped snake from Gujarat. Given that the specimen was not available to the authors, the exact identity of the striped snake remained in question. Over the years the second author (RV) recorded this morph from several localities in the state and had two specimens sent to him from two other localities. Recently one of us rescued an adult male specimens of the striped form depicted in Fig 1 by Vyas & Patel [18] and the specimens from RV enabled us to investigate its phylogenetic position using morphological as well as molecular data. Results from molecular data show that the snake belongs to a clade arid snakes with *Platyceps* Blyth, *Eirenis* Jan, *Spalerosophis* Jan, *Macroprotodon* Guichenot and *Lytorhynchus* Peters etc. The snake however greatly differs morphologically as well as genetically which warrants erection of a new genus to accommodate the new species from Gujarat.

In the present communication, we describe the snake from Gujarat as a new species and propose a new genus to accommodate the striped form of *Coronella* [18]. Data from molecular dating allows us to discuss biogeography of the new species.

## Material and Methods

### Ethics statement

The present study was conducted with appropriate permissions granted by the Forest Department of Gujarat state permit no. WZP/5585/22/C/590-92/3-8-1990 and B/WPS/8/9388-92/2013-14 in accordance with the Indian Wildlife Protection Act 1972. Furthermore, two of the type specimens were found dead in unprotected areas and the species collected is not listed in IUCN Redlist or by CITIES. The holotype was collected alive and was euthanized using ketamine treatment following AVMA guidelines [20] and did not require approval by an ethics committee.

### Molecular phylogeny and analysis

Genomic DNA was extracted using Qiagen DNeasy^TM^ Tissue kits followed by amplification of three mitochondrial (12s & ND4 for two specimens & 16s for only the one specimen) and one nuclear genes (c-mos for two specimens) following Pyron et al. [8]. Primers used for amplification of target DNA were based on previous studies Schätti & Utiger [16] and Pyron et al. [8]. PCR reaction of 10μl were set containing 4μl Qiagen^TM^ Taq PCR Master Mix, 3μl water, 0.5μl of each forward and reverse primer and 2μl of genomic DNA were set on a temperature gradient for temperature optimization using a Eppendorf Mastercycler Nexus GSX1. PRC products were cleaned with QIAquick PCR Purification Kit and were sequenced with a 3730 DNA Analyzer. Sequences of protein coding genes were aligned using MACSE [21] to check for frameshifts and codons and were concatenated with non-coding gene sequences in Geneious R6 v.6.18. [22]. For elucidating phylogenetic relationship of the snake, we performed a Maximum Likelihood analysis of 410 species of snakes based on ~570bp of cmos gene which showed that the snake is a member of the arid clade containing the genera *Platyceps*, *Macroprotodon*, *Lytorhynchus* and *Hemerophis* etc. In order to ascertain this, we concatenated data of five genes (nuclear: cmos ~570bp; mitochondrial: cytb 1118bp, ND4 ~676bp, 12s ~343, 16s 338bp) and performed a Maximum Likelihood analysis in RAxML v. 8.0.0 [23] for 48 taxa listed in “S1 Table”. Bayesian analysis was performed with BEAST v.1.7.3 [24] with concatenated cmos and cytb sequences for Bayesian tree with posterior probability support. To estimate the age of divergence of the new genus, we included taxa that could be used for node calibrations. Calibrations follow Nagy et al. [2] excluding rooting with a mammal. The tree output file was visualized and edited in FigTree v1.3.1. Sequences of the new species have been submitted to GenBank with the following accession numbers: KR819917–KR819923. Ancestral area reconstruction was performed on Bayesian tree with RASP [25]. Each node was coded with their present distribution following zoogeographic zones of the world following Holt et al. [26].

### Morphology and other analysis

Specimens were fixed in 4% formalin and later stored in 70% ethanol and is deposited in the collection of the National Centre for Biological Sciences, Bangalore (NCBS) and Bombay Natural History Society, Mumbai (BNHS). Measurements were taken with a Mitutoyo^TM^ digital caliper to the nearest 0.01mm and total length was measures using a thread. Ventral scales were counted following Dowling [27] and scale reduction method follows Dowling [6] with modifications needed for representing vertebral scale reductions. To describe the position of dorsal scale row reductions/increase, total ventral scales have been converted in percentage and the reduction/increase is not presented at the actual ventral number but in percentage. Maxillary and palatine bones were dissected from the holotype to study the number and nature of dentition and were observed under a Leica^TM^ S8APO stereo-binocular microscope. Images were taken with the help of a Canon 70D mounted with a Canon 100mm macro and illuminated with the help of a two Canon 430EXII speedlight. Illustrations were prepared by RVS. Morphological data for related genera were obtained from Smith [7], Schätti & Trape [15], Schätti & Monsch [14], Schätti et al. [17] and Schätti & Utiger [16]. Sea level rise imagery were taken from http://calculatedearth.com/ to ascertain isolation of Saurashtra form mainland India. These images were recreated in Adobe Photoshop CS6 and QGIS for publication.

### Nomenclature acts

The electronic edition of this article conforms to the requirements of the amended International Code of Zoological Nomenclature, and hence the new names contained herein are available under that Code from the electronic edition of this article. This published work and the nomenclatural acts it contains have been registered in ZooBank, the online registration system for the ICZN [28]. The ZooBank LSIDs (Life Science Identifiers) can be resolved and the associated information viewed through any standard web browser by appending the LSID to the prefix "http://zoobank.org/". The LSID for this publication is: urn:lsid:zoobank.org:pub: E1E0E289-527A-4506-985B-85159BC367E5. The electronic edition of this work was published in a journal with an ISSN, and has been archived and is available from the following digital repositories: PubMed Central, LOCKSS.

## Results

### Systematics

#### *Wallaceophis* gen. nov. Mirza, Vyas, Patel & Sanap, 2016

urn:lsid:zoobank.org:act:43CA682D-4EE0-4653-95B6-6F4E0C2FAEDE

#### Type species

Wallaceophis gujarateneis **sp. nov.**

#### Diagnosis

**Fig 2:**
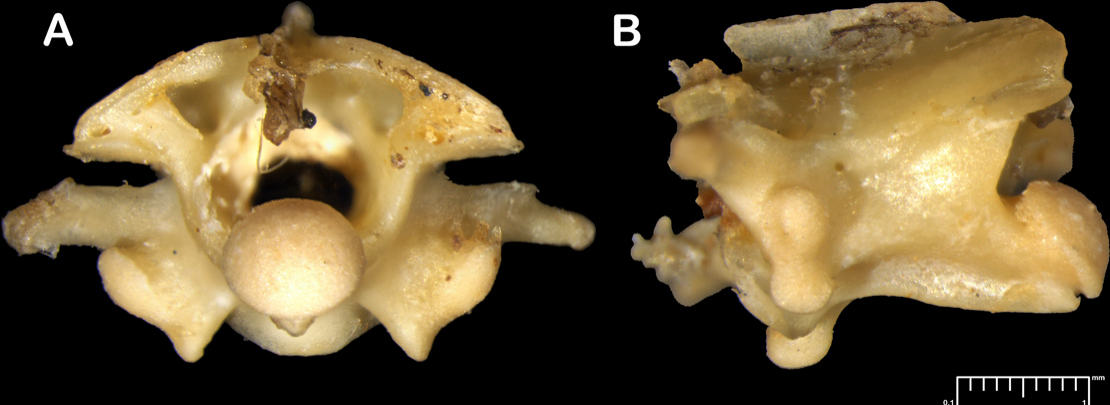
*Wallaceophis* **gen. et.** *gujaratensis* **sp. nov.** NCBS HA-106 vertebrae from mid-body, (A) anterior view, (B) lateral view.

A medium sized snake in relation to members of the family measuring SVL 250–930 mm differing from most colubrid genera in lacking hypapophyses on posterior dorsal vertebrae (Fig 2) and in bearing nine maxillary teeth and the posterior-most teeth are subequal, nine palatine teeth. Dorsal scale reduction characterized by vertebral reductions, increase of scale rows posterior to neck, a single lateral reduction at midbody and regular vertebral reductions in posterior half of the body. Rostral not visible from above, a small presubocular present. Eight supralabials, fourth and fifth in contact with orbit, anal undivided, 215–216 ventrals, 51–54 subcaudals, hemipenis subcylindrical, spinose throughout and 3–4 dorsal scale row wide black longitudinal stripe running from the post nasal to the tail tip on each side on a wheat colored dorsum.

*Wallaceophis* **gen. nov.** may be distinguished from most members of the family Colubridae in lacking hypapophyses on posterior dorsal vertebrae. This condition is present in racers and whip snake of the genera *Platyceps*, *Hemorrhois*, *Spalerosophis*, *Hemerophis*, *Dolichophis*, *Hierophis*, *Eirenis*, *Orientocoluber*, *Coluber*, *Macroprotodon*, *Bamanophis* and *Lytorhynchus*. *Wallaceophis* **gen. nov.** differs from these genera in bearing unique vertebral dorsal scale reductions (vs. lateral reductions in *Platyceps*, *Hemorrhois*, *Hemerophis*, *Dolichophis*, *Hierophis*, *Eirenis*, *Orientocoluber*, *Coluber*, *Macroprotodon*, *Bamanophis* and *Lytorhynchus*); nine maxillary teeth (vs. 15–17 in *Spalerosophis*, 14–19 in *Platyceps*, 13–16 in *Hemorrhois*, 17–20 in *Hemerophis*, 16–18 in *Hierophis*, 16–26 in *Eirenis*, 15–19 in *Bamanophis*); presubocular present (vs. absent in *Macroprotodon*, *Orientocoluber*, *Bamanophis*). The new genus is closely related to the genus *Lytorhynchus* based on ~3047bp of nuclear and mitochondrial gene sequences however differs from it in having vertebral dorsal scale reduction (vs. lateral in *Lytorhynchus*); nine palatine teeth (vs. 3–5 in *Lytorhynchus*).

#### Etymology

The proposed generic name is a compound of two words, the first being a patronym honoring Alfred Russel Wallace for his pioneering work on biogeography and for co-discovering the theory of natural selection with a suffix ‘*ophis*’ (όφις) meaning snake in Greek. Gender of the proposed generic name is masculine.

#### *Wallaceophis gujaratensis* sp. nov. Mirza, Vyas, Patel, Maheta & Sanap, 2016

urn:lsid:zoobank.org:act:6616529A-8EC2-4606-878C-253F2CD0E6B1

(Figs 1–6)

**Fig 3:**
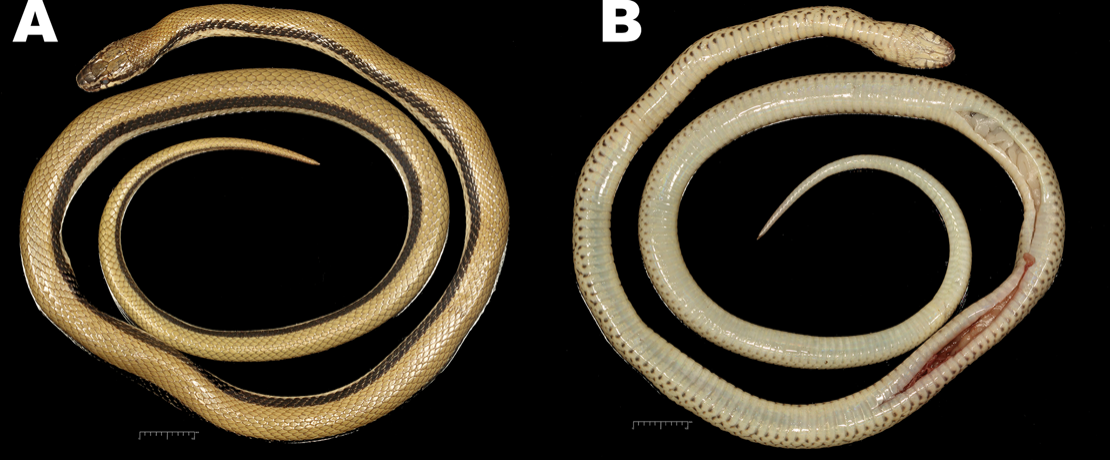
*Wallaceophis* **gen. et.** *gujaratensis* **sp. nov.** holotype male NCBS HA-105 (A) dorsal view, (B) ventral view.

**Fig 4:**
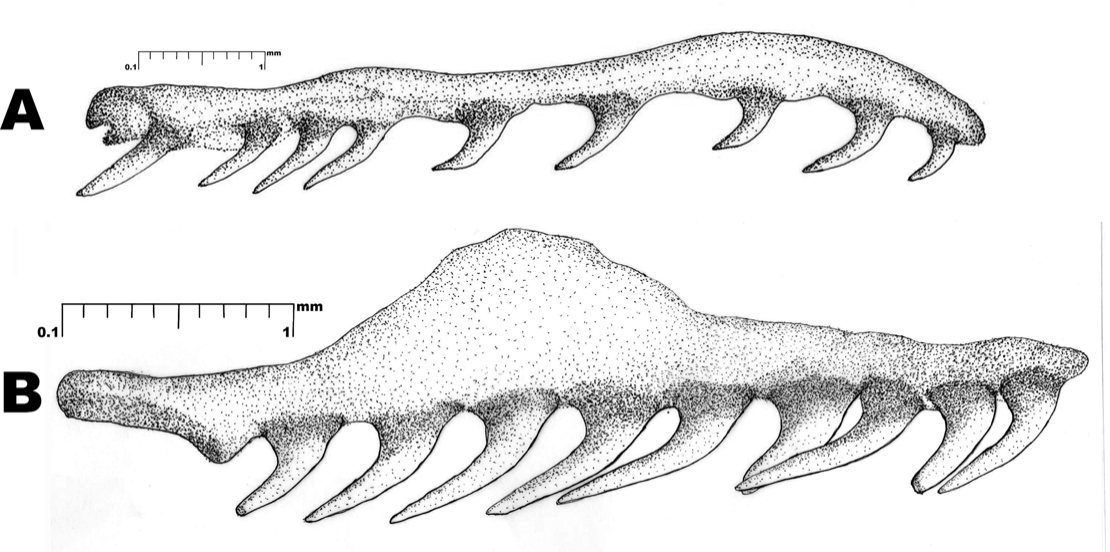
*Wallaceophis* **gen. et.** *gujaratensis* **sp. nov.** holotype male NCBS HA-105, (A) right maxilla, (B) right palatine.

**Fig 5:**
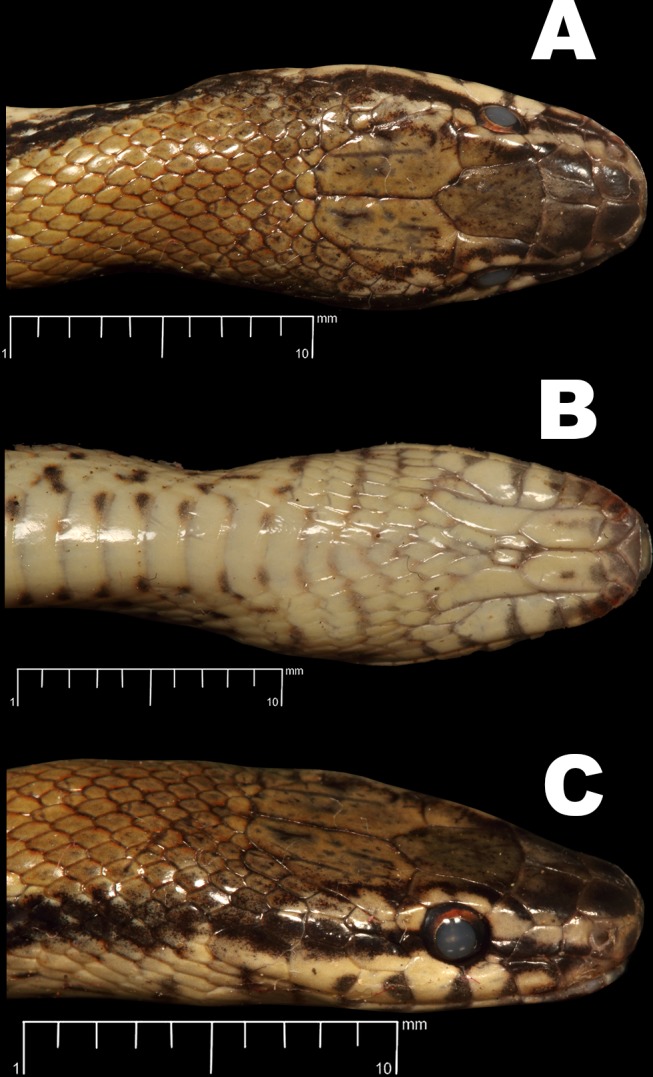
*Wallaceophis* **gen. et.** *gujaratensis* **sp. nov.** holotype male NCBS HA-105 head, (A) dorsal view, (B) ventral view & (C) lateral view.

**Fig 6:**
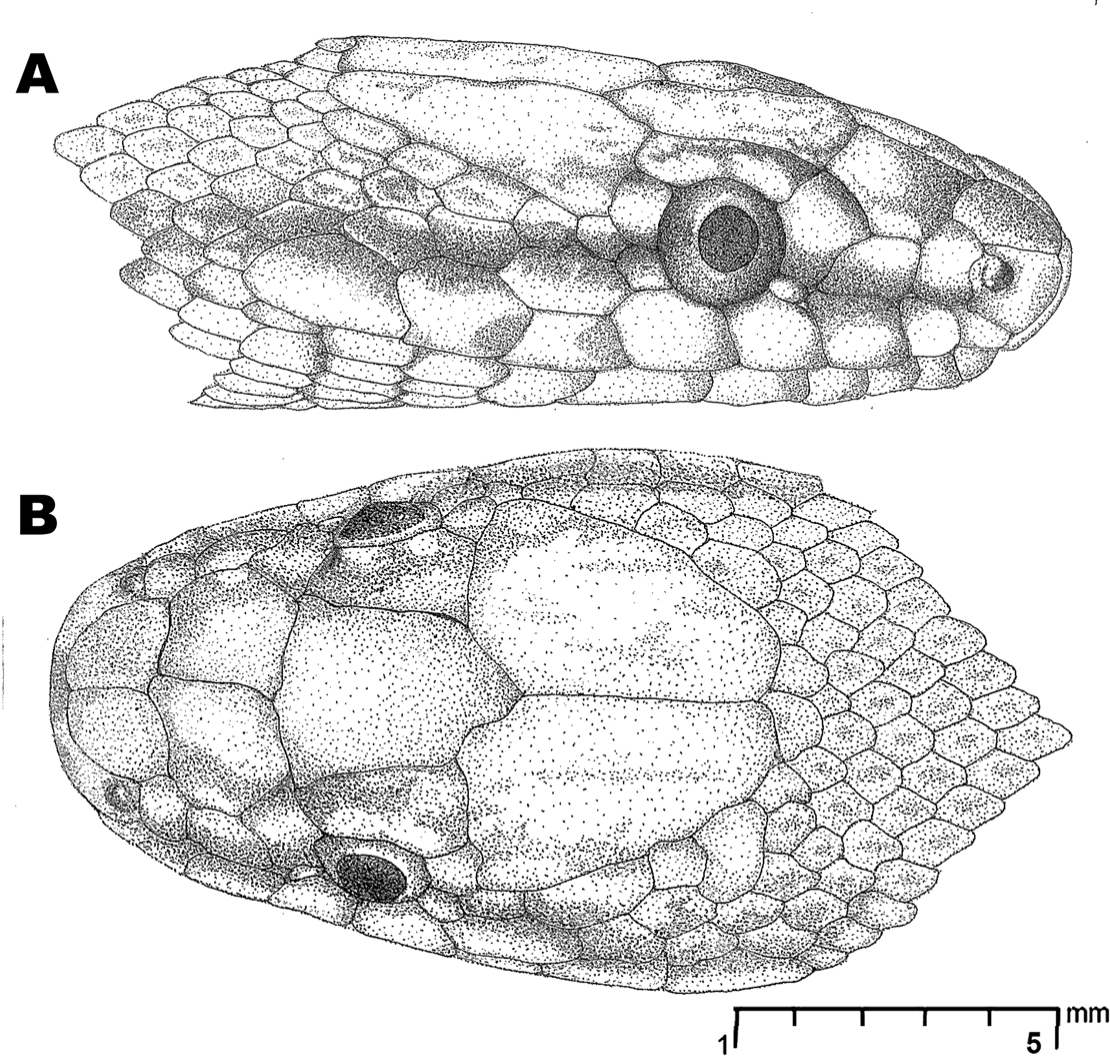
*Wallaceophis* **gen. et.** *gujaratensis* **sp. nov.** holotype male NCBS HA-105 head illustration showing scalation, (A) lateral view, (B) dorsal view.

#### Holotype

male, NCBS HA-105, collected from Khengariya village, Viramgam taluka, Ahmedabad district, Gujarat state, India (23.0217946 N, 72.0217584 E, altitude 21m) by Jaydeep Maheta on 24th July 2014.

#### Paratypes

female BNHS 3503, collected form near Amreli, Amreli district, Gujarat state, India by Viral Joshi on 20^th^ March 2013.

#### Other material

NCBS HA-108, an unsexed, damaged individual from Malod, Wadhwan Taluka, Surendranagar District, Gujarat (22.684461 N, 71.6501 E) by Kartik Upadhayay on 12^th^ April 2014.

#### Etymology

The specific epithet refers to Gujarat state in western India where the new species was discovered.

#### Description of holotype male NCBS HA-105

The specimen is in good condition preserved in a coil with a mid-ventral longitudinal incision at mid-body and another under the tail (Fig 3A and 3B).

Dorsally wheat coloured with 3–4 dorsal scale rows wide black longitudinal lateral stripe from post nasal till the tip of tail (Fig 1). Head dark wheat coloured dorsally with diffused dark spots/patches, internasals and prefrontals in a darker shade; head laterally creamish with a brown stripe from nasal region all through the orbit merging with the lateral stripe along the body. Supraoculars with dark brown diffused patches on anterior portion. Frontal heavily dotted with dark brown spots and diffused markings. Parietals with short longitudinal dark brown lines present. Colour of eye golden with an orange ting (Fig 1). Venter and labials creamish white; labials bordered with grayish colour and each ventral scale with a brown spot on each anterior corner. Ventrolateral portion of snake with fairly equidistant black spots, distinct and aggravated anteriorly and diffused on posterior part of the body. Specimen slightly paler in preservative (Fig 3A and 3B).

Head short, 14.83 mm comprising 2.95% of total length; high, 3.99 mm, with steeply domed snout in lateral view. Snout tapering to blunt, rounded tip in dorsal view (Figs 3E and 4A). Triangular rostral, not visible from dorsal view; wider than deep. Nostrils large, crescent shaped, between two sub-pentagonal nasal scales. Paired internasals large, slightly wider (1.25) than long (1.23); smaller than prefrontals. Prefrontals, longer (1.72) than wide (1.70); frontal bell shaped, 2.74 at the widest anterior border, median length 3.55 and 1.97 posterior width at suture with supraocular. Parietals 4.59 long, 2.88 at its widest anterior, 0.88 at its posterior border (Figs 3C and 4B). Temporals 2 + 2 on the left/ 2+1+2 on the right, subequal in size, posterior one inserts deeply between supralabial fifth and sixth. Eight to nine nuchal scales, larger than adjesant dorsal scales, bordering parietals. Supraocular slightly smaller than preocular; preocular large, deeper than wide. Loreal wider (1.34) than long (0.84). Two postoculars, upper one larger. Eye circular, 1.55 diameter with a rounded pupil. Eight supralabials, fourth and fifth in contact with orbit. First supralabial very small and, apart from second supralabial, contacts only rostral and nasal (Figs 5A–5C, 6A and 6B). Second supralabial small, contacting nasal, loreal and first and third supralabials. Third subequal, fourth and fifth supralabial scales much larger, taller than long. Fourth supralabial in contact with presubocular, third and fifth supralabials and scarcely touching loreal. Fifth supralabial the tallest and eighth longest.

Mental short, broad, triangluar (Fig 5B). Anterior four infralabials short and thin, fifth onwards larger. Fifth and sixfth infralabials much larger, smaller in size as each of a pair of posterior genials. Anterior genials twice as long (3.08) as wide (1.50), posterior genials 3.42 long and 0.98 wide. Posterior pair separated by two to three rows of scales (Fig 5B).

Maxilla with nine teeth lacking a distinct diastema (Fig 4A). The posterior-most one slightly enlarged or subequal. First five teeth equidistant from each other, followed by a cluster of three teeth and a single one separated apart. Palatine with nine teeth placed continuously lacking diastema (Fig 4B).

Body subcylindrical, ventral surface a little flattened. Dorsal scales formula as in Table 1, up to posteriormost ventral. Dorsal scale row reduction unique, with only two lateral reduction and a series of vertebral increase and reduction. First lateral reduction in less <1% of the ventral scales with a single vertebral addition at 14% which is reduced by a vertebral reduction at 56%. The second lateral reduction is observed at 58.7% followed by four vertebral reductions at 65.7%, 67%, 94% & 96%. Dorsal scales imbricate, regularly arranged, evenly sized, each with an apical pit. All body scales smooth and glossy, lacking keels. Ventral scales 216 in number. Anal shield undivided, similar in size to last ventral scale, its posterior margin overlaps four small, irregular scales on right and five on left, in addition to pair of larger subcaudals medially. Subcaudals paired, 54 in number. Tail terminates in a sharp tapering apical spine. Total length 501.4 mm, tail length 60.9 mm, tail/total length ratio 0.138. Anteriorly with 11 to 13 dorsal scale rows, reducing to about six at mid tail, four surrounding base of terminal spine.

**Table 1:** Dorsal scale row reduction formula of *Wallaceophis* gen. et. *gujaratenesis* sp. nov. holotype male NCBS HA-105.

$25\frac{4+5(3)}{4+5(2)}23\;12=12+13\left(31\right)\;24\;10+11\left(123\right)\;23\frac{4+5(127)}{4+5(127)}21\;\text{-}10\left(142\right)\;20\;\text{-}11\left(145\right)19\;\text{-}10\left(204\right)\;18\;\text{-}9\left(208\right)\;17(216)$

Hemipenis extending to the 16^th^ subcaudal plate. Unilobed, aspinose at base. Small spines start 1/3^rd^ of its length from base which gradually increase to large spines directed downwards.

#### Variation

All examined and referred specimens match with the holotype and differ in the following respect- SVL range 253–930mm (n = 9); ventrals 215 (BNHS 3503); subcaudals 51, of these five entire/not divided.

#### Suggested common name

Wallace’s striped snake/ Wallace’s racer

### Natural History & Distribution

**Fig 7:**
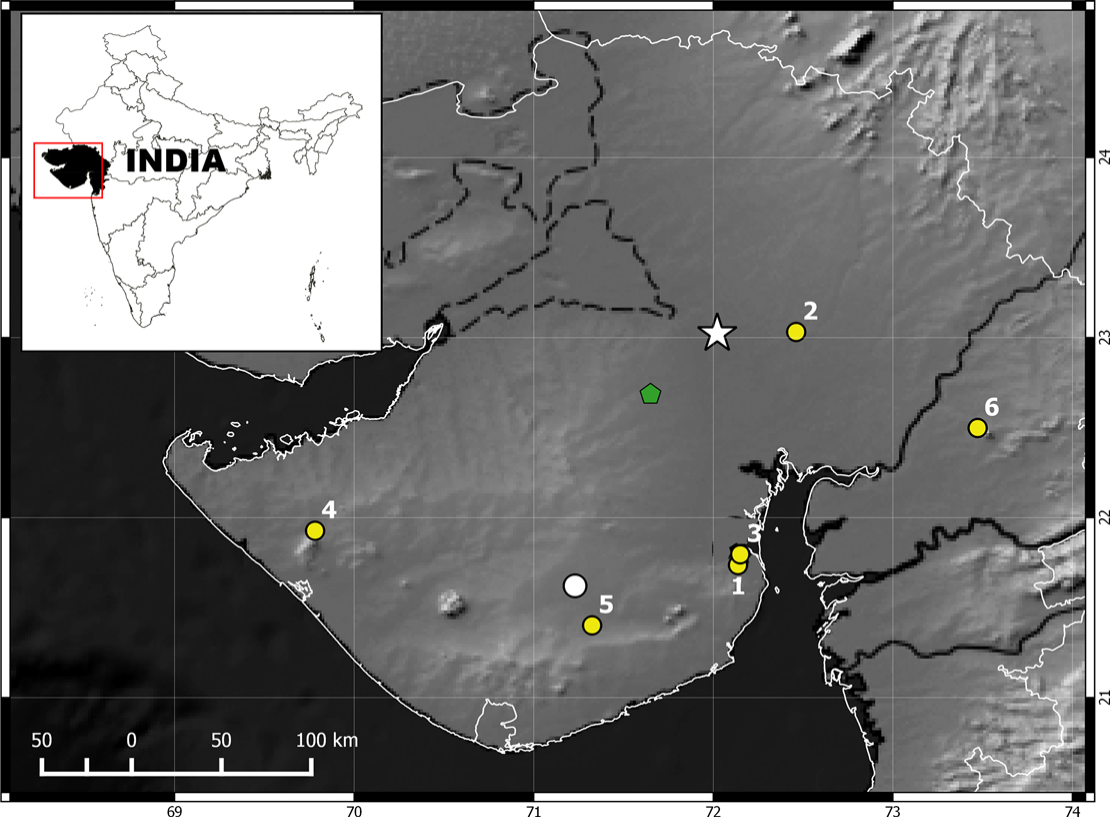
Map showing type locality and known records for *Wallaceophis* gen. et. *gujaratensis* sp. nov. Locality indicated by a white star for holotype NCBS HA-105 and white circle for paratype BNHS 3503, green polygon for NCBS HA-108; localities indicated by yellow circles are based on photographic evidence. Inset map showing position of Gujarat state in western India highlighted in black. 1- Sagwadi, Bhavnagar; 2- Bopal, Ahmedabad; 3- Old Port, Bhavnagar; 4- Bhanvad, Jamnagar; 5- Saladi, Amareli; 6- Halol, Panchmahal.

The type specimen was collected from a manmade water hole near an irrigation canal along with a few juveniles of *Xenochropis piscator*. The species appears to be diurnal as it was collected at *ca*. 11:15 hours. The type locality, Khengariya village, is situated in the dry plains of central-western region of Gujarat state. According to Champion and Seth [29] the type locality falls under Desert thorn forest. The floral composition of this area is made up of *Acacia senegal*, *Acacia leucophloea*, *Euphorbia neriifolia*, *Capparis* spp., *Zizyphus* spp., etc. The region falls under the drier parts of the country. The annual precipitation is 838mm. Majority of the precipitation occurs during the months of July and August. The temperature varies from as low as 12^°^C during winter and as high as 43^°^C during the hot summer days. These conditions create a harsh environmental condition for any life form living in this area. The snake was immersed in water to wash it upon which the snake dived to the base of the bucket and remained submerged for about five minutes. The holotype was also found in water suggesting that the snake might prefer areas in proximity to water sources. While photographing the snake, the snake made attempts to dig into the substrate which suggest that the snake is fossorial in nature. An individual retained in captivity was offered a *Hemidactylus* sp. which was readily accepted. We have also been able to collect the secondary information about the species’ habits and habitat from local ‘snake rescuers’ and wildlife photographers on the basis of colored images/photographic evidences (Table 2). This yielded information denotes that the species inhabits other parts of the state too, including the holotype, paratype and the specimen NCBS HA-108 (Fig 7). The information from various sources and collection sites of specimens shows that species is distributed in four different sub biotic land regions as 4B1Saurashtra Plateau, 4B2-Bhal, 4B4 Plains and 4B5Plains of Gujarat and this entire land mass further falls in 4B Semi-Arid Gujarat-Rajputana Provinces as per the Biogeographic Zone Classification of Rodgers and Panwar [30]. For a summary of distribution localities, see Table 2.

**Table 2:** Distribution localities of *Wallaceophis* gen. et. *gujaratenesis* sp. nov. based on specimens and/or photographic evidence.

	Date	SVL	Locality	GPS co-ordinates	Habitat	Remark
1	**–**	320	Sagvadi, Bhavnager	21°44'8.73"N, 72° 8'15.60"E	Human habitation	Specimen examined
2	28/06/2006	**–**	Bopal, Amadavad	23° 1'49.94"N, 72°27'42.61"E	Human habitation	Photographs examined
3	26/05/2006	**–**	Old port, Bhavanagar City	21°47'43.63"N, 72° 9'0.20"E	Industrial area	Photographs examined
4	19/04/2007	**–**	Bhanvad, Jamnagar	21°55'34.28"N, 69°46'55.34"E	Human habitation	Photographs examined
5	15/09/2010	320	Saladi, Amareli	21°24’1.59”N, 71°19’30.65”E	Human habitation	Photographs examined
6	29/09/2010	450	Saladi, Amareli	21°24’1.59”N, 71°19’30.65”E	Human habitation	Photographs examined
7	20/11/2010	570	Saladi, Amareli	21°24’1.59”N, 71°19’30.65”E	Human habitation	Photographs examined
8	20/11/2010	590	Saladi, Amareli	21°24’1.59”N, 71°19’30.65”E	Human habitation	Photographs examined
9	20/03/2013	253	Nr. Amareli	**–**	Road-kill	BNHS 3503
10	11/03/2013	930	Halol, Panchmahal	22°29'48.88"N, 73°28'20.59"E	Industrial area	**–**
11	27/06/2014	400	Vaghela Mine Wadhavan	22°39'31.00"N, 71°39'48.99"E	Found in bird’s foods	Photographs examined
12	12/04/2014	*ca*. 350	Nr, Malod, Wadhavan	22°41'4.06"N, 71°39'0.36"E	Found in bird’s foods	NCBS HA-108
13	24/07/2014	501	Khengariya, Viramgam	23°1'18.462"N, 72°1'18.3288"E	Rescued	NCBS HA-105

## Discussion and Conclusion

**Fig 8:**
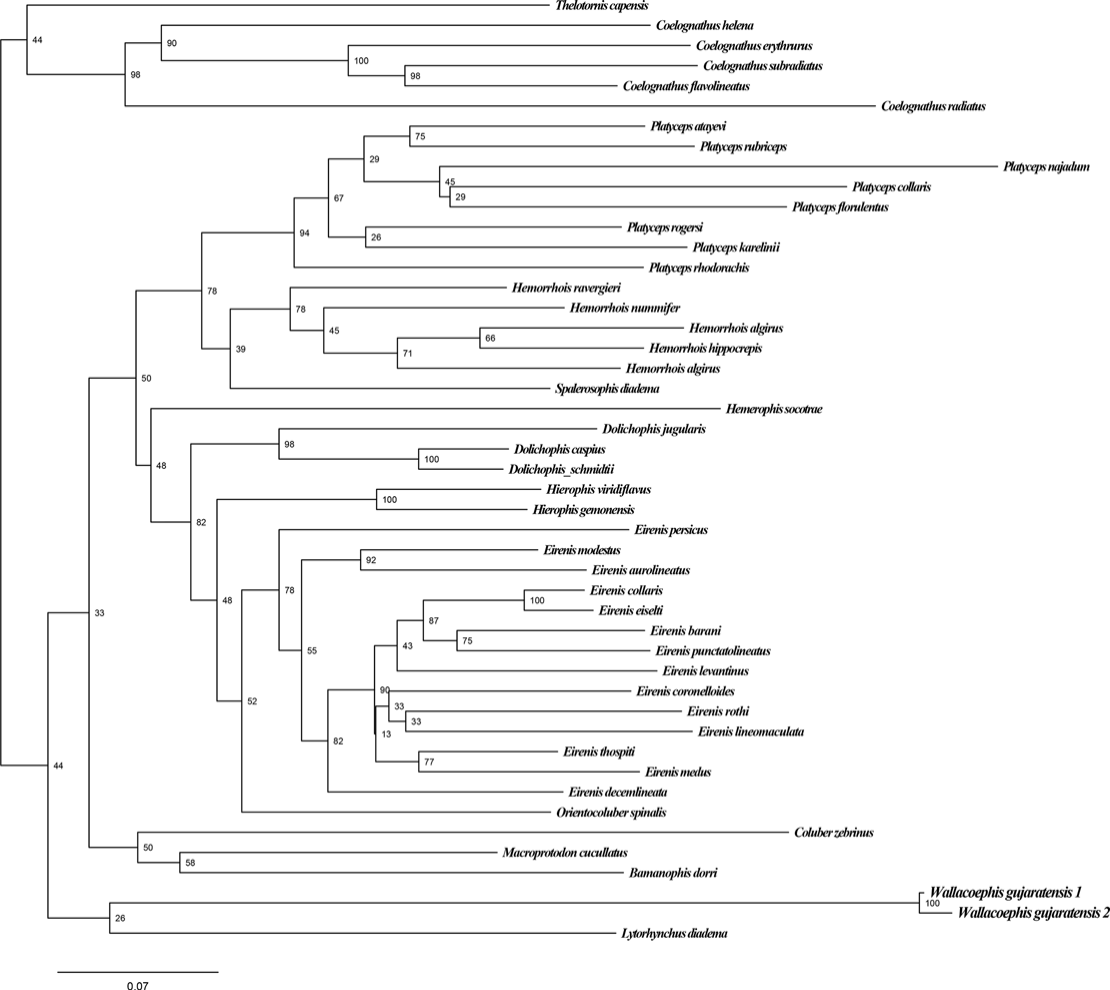
Maximum Likelihood phylogeny (cmos, cytb, nd4, 12s, 16s) with GTR +G model of selected snakes of the subfamily Colubrinae. Numbers at nodes represent likelihood bootstrap support with 1000 replicates. Out-group excluded.

Phylogenetic analysis based on a total of 3047bp of concatenated nuclear and mitochondrial genes shows that *Wallaceophis* **gen. nov.** is a member of a clade of arid snake species within Colubrinae containing the genera *Hemorrhois*, *Platyceps*, *Hierophis*, *Hemerophis*, *Eirenis*, *Dolichophis*, *Orientocoluber*, *Bamanophis*, *Macroprotodon* and *Lytorhynchus* (Fig 8). The relationships recovered from our analysis are congruent with those of Pyron et al., [8,9,31]. *Wallaceophis* **gen. nov.** is genetically most similar to the genus *Lytorhynchus* with an uncorrected pairwise sequence divergence of 21.5% for nuclear cmos gene and is recovered as a sister taxa of the new genus with a deep divergence. The new genus shows 23–36% uncorrected pairwise sequence divergence for nuclear cmos gene from other genera of the clade (Table 3). Our analyses are preliminary and must however be confirmed after incorporation of more taxa of the genus *Lytorhynchus* as well as data for additional nuclear genes. Based on morphology *Wallaceophis* **gen. nov.** can be readily distinguished from all members of the old world racers in have a unique dorsal scale row reduction pattern in addition to bearing fewer maxillary teeth.

**Table 3:** Uncorrected pairwise sequence divergence (%) between *Wallaceophis* gen. et. *gujaratenesis* sp. nov. and old world racer and whip snake genera for the nuclear gene cmos. Divergence between two specimens of *Wallaceophis* **gen. et.** *gujaratenesis* sp. **nov.** is 2%.

Genera	Sequence divergence
*Bamanophis*	36
*Dolichophis*	30–32
*Eirenis*	25–32.2
*Hemerophis*	28.6
*Hemorrhois*	23.3–30.4
*Hierophis*	23.3–32.2
*Hierophis*	23.3–34
*Lytorhynchus*	21.5
*Macroprotodon*	28.6
*Orientocoluber*	27
*Platyceps*	25–28.6
*Spalerosophis*	28.6

Molecular dating reveals that the split between the genus *Lytorhynchus* and *Wallaceophis* **gen. nov.** took place around early Miocene about 17.1 MYA (11.08–23.05 MYA, S1 Fig) which is when the Indian plate was well connected to Asia [32]. The genus *Lytorhynchus* is distributed across northern Africa through Sub-Saharan region up to the Indian state of Rajasthan and members of this genus are sand dwelling species. A single species of the genus *Lytorhynchus* is reported from only three localities in Rajasthan [33]. The new monotypic genus on the other hand is restricted to western and central parts of Gujarat state.

**Fig 9:**
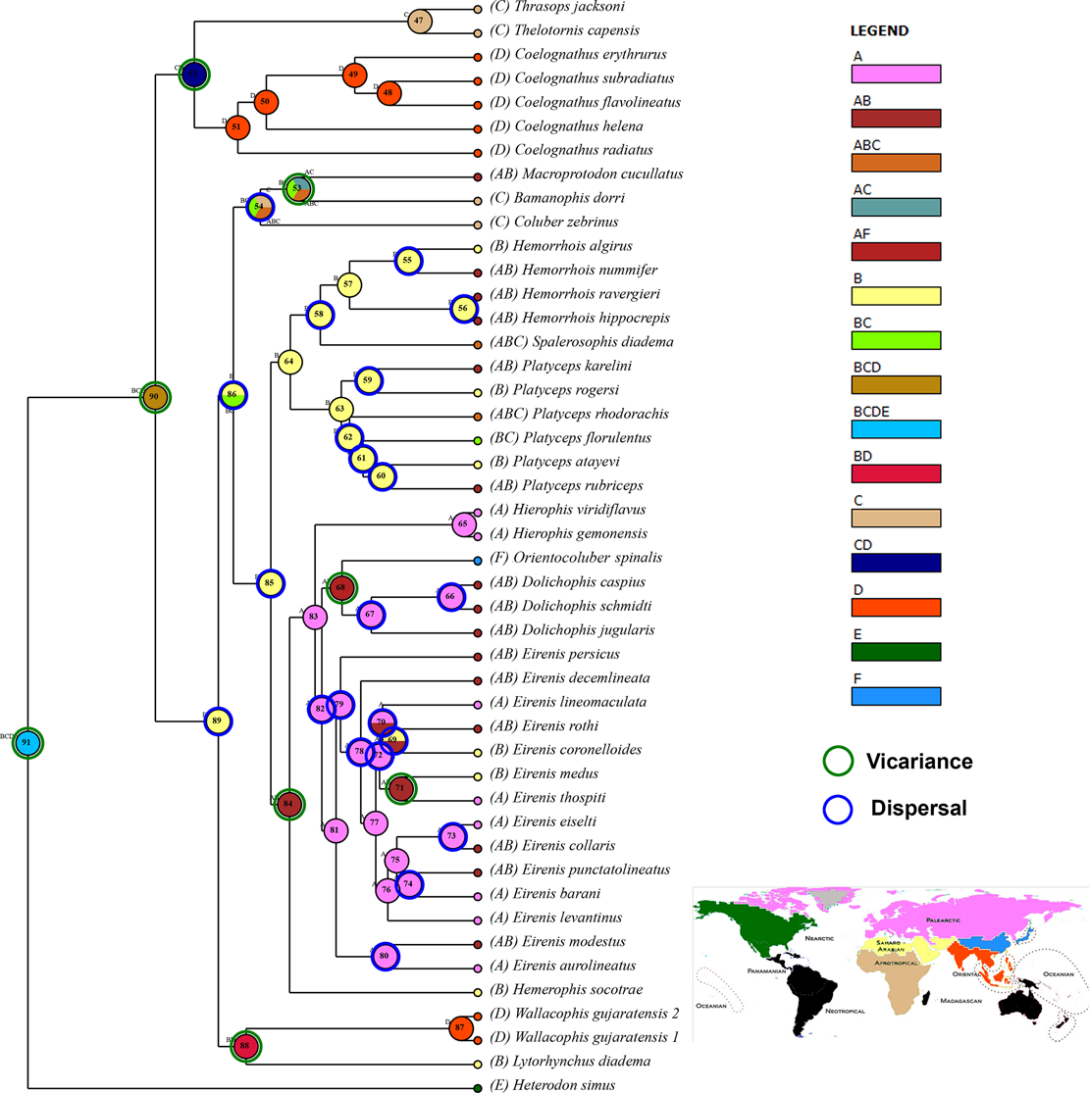
Ancestral area cladogram selected snakes of the subfamily Colubrinae. Inset map of world showing zoogeographic regions of the world modified after Holt et al. (2013). Regions of the World coded as follows: ‘A’ Palearctic, ‘B’ Saharo-Arabian, ‘C’ Afro-Tropical, ‘D’ Oriental, ‘E’ Nearctic, ‘F’ Sino-Japanese.

**Fig 10:**
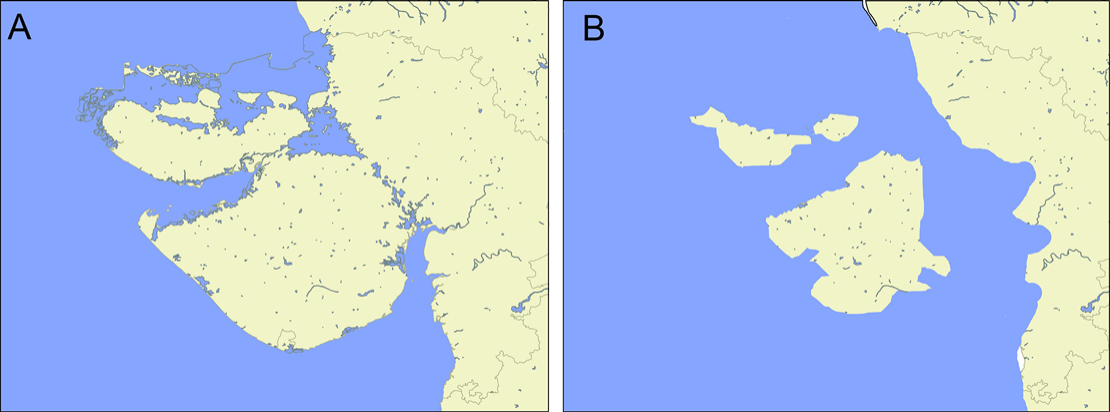
Map showing the western Indian state with two different sea levels highlighting Saurashtra as a functional island (A) 0m (present), (B) 15m (prior to rise of Himalayas).

A vicariance hypothesis in which speciation resulted due to long isolation on Saurashtra then an island during early Miocene for *Wallaceophis* **gen. nov.** is proposed based on dispersal and vicariance analysis (Fig 9). Ancestral lineage likely dispersed from Saharo-Arabian region through Trans-oceanic dispersal and colonized Saurashtra. Rising Himalayas resulted in ~50m of sea level drop and hence simulation of sea level rise clearly show that Saurasshtra was an island and hence we propose a Trans-oceanic dispersal and not by land as Saurashtra was bound by the sea at least until early Holocene. Colonization by oceanic dispersal seems more plausible as Saurashtra was surrounded by water (Gaur et al. 2013) prior to the rise of Himalayas. After Indian plate collided with Eurasia, uplift of Himalayas between 35–20MYA and Antarctic ice sheet growth 10.5 MYA [34,35] resulted is seal level drop to ~50m (Condie, 1997) creating land connections between Saurashtra and mainland India (Fig 10). Ancestral area reconstruction analysis indicates that the ancestor of *Lytorhynchus* and *Wallaceophis* **gen. nov.** had a Saharo-Arabian origin which dispersed into the oriental region (Fig 9). The fact that islands are known to harbor peculiar endemic radiation and have been seen across taxon on large as well as small islands [36,37]. Saurashtra too might certainly be home to several more endemic radiation of across other taxa which needs to be explored as a morphologically distinct taxa like a snake remained undocumented hitherto despite numerous authors made attempts to produce a comprehensive compilation on Indian snakes in the last fifty years.

Oceanic dispersal has largely been considered as a rare event and an explanation of last resort however it seems more common and plausible explanation in many taxa especially plants, reptiles and amphibians [38]. Many studies have shown that several lineages of snakes have dispersed via oceanic dispersal [2,39,40]. Colonization of biota in India has largely been attribute to (i) rafting Indian plate carried biota from Africa/Madagascar, (ii) invasion of Asian biota after Indian made contact with Asia however the possibility of oceanic dispersal shaping India’s biota has not been explored and provides new dimensions for research. Rise of Himalaya’s has largely influenced climatic conditions in India as well as its biota as highlighted in the present work as well as previous workers [41]. Sea level rise and drop reshape landmass which greatly influence their biota and these fluctuations in sea levels due to Himalayan uplift appears to be an interesting aspect for biogeographic investigations and research. In addition to this, Saurashtra being an island provides an avenue for research across taxa to explore effects of long term isolation of biota.

India is home to ~280 species of snake which highlight the high diversity of serpents in the country. After publication of the Fauna of British India series by Smith (1935, 1943), it appeared that the reptilian fauna of India is well documented. However, in the recent years, numerous new species of reptiles have been described from the country highlighting the need for more dedicated surveys across the country (see [42–47]).

Gujarat, the western most state of India, is unique in terms of habitat diversity. Most of the major mountain ranges of Peninsular India, such as Aravalli, Vindhya, Satpura and Western Ghats have a terminus in Gujarat. The varied geology and topography of these hills is coupled with the extensive xeric regions that are contiguous with those of Rajasthan and Pakistan, coupled with changing landscape connections due to fluctuating sea levels results in high habitat diversity, which supports a unique and diverse biota. However, except for few surveys, forests of Gujarat are herpetologically unexplored. Discovery of a new gecko *H*. *gujaratensis* [48] and other recent additions of herpetofauna of Gujarat [49] indicate that dedicated surveys across the state may yield additional herpetological discoveries from Gujarat. Discovery of *Wallaceophis gujaratensis* **gen. et. sp. nov.** further adds to the growing knowledge of the high degree of unique and endemic herpetofauna in the country (see [50–53]) and also brings to light the poor nature of herpetofaunal documentation in the country. Systematics of Indian snakes is largely unattended and is in need of revision after incorporating molecular and morphological data which will inevitably result in discovery of many more diverse and such distinct lineages.

## Supporting Information

S1 Fig Bayesian timetree of selected snakes of the family Colubridae showing split between *Wallaceophis* gen. nov. and related genera. Numbers at node indicate Bayesian posterior probabilities and blue bar indicates 95% HPD.
(TIF)

S1 Table Genbank accession numbers of sampled taxa for analysis. (DOCX)

## References

[1] Lawson R, Slowinski J, Crother B, Burbrink F (2005) Phylogeny of the Colubroidea (Serpentes): new evidence from mitochondrial and nuclear genes. Mol Phylogenet Evol 37: 581–601. 16172004 10.1016/j.ympev.2005.07.016

[2] Nagy ZT, Joger U, Wink M, Glaw F, Miguel V (2003) Multiple colonization of Madagascar and Socotra by colubrid snakes: evidence from nuclear and mitochondrial gene phylogenies. Proc R Soc B Biol Sci 270: 2613–2621. 10.1098/rspb.2003.2547 PMC1691547 14728785

[3] Uetz P, Hošek J (2015) The Reptile Database. Available: http://www.reptile-database.org. Accessed 6 July 2015.

[4] Wallach V, Williams K, Boundy J (2014) Snakes of the World: A catalogue of living and extinct species. London: Taylor & Francis Group. 1257 p.

[5] Zug G, Vitt L, Caldwell J (2001) Herpetology: an introductory biology of amphibians and reptiles. San Diago: Academic Press. 645 p.

[6] Dowling H (1951) A proposed method of expressing scale reductions in snakes. Copeia 2: 131–134.

[7] Smith MA (1943) Fauna of British India, Ceylon and Burma, including the whole of the Indo-Chinese Sub-region Reptilia and Amphibia. Vol. 3. Serpentes. London: Taylor and Francis. 583 p.

[8] Pyron RA, Burbrink FT, Colli GR, Vitt ANM de OLJ, Kuczynski CA, Wiens JJ (2011) The phylogeny of advanced snakes (Colubroidea), with discovery of a new subfamily and comparison of support methods for likelihood trees. Mol Phylogenet Evol 58: 329–342. 10.1016/j.ympev.2010.11.006 21074626

[9] Pyron RA, Kandambi HKD, Hendry CR, Pushpamal V, Burbrink FT, Somaweera R (2013) Genus-level phylogeny of snakes reveals the origins of species richness in Sri Lanka. Mol Phylogenet Evol 66: 969–978. 10.1016/j.ympev.2012.12.004 23261713

[10] Utiger U, Helfenberger N, Schätti B, Schmidt C (2002) Molecular systematics and phylogeny of Old and New World ratsnakes, *Elaphe* Auct., and related genera (Reptilia, Squamata, Colubridae). Russ J Herpetol 9: 105–124.

[11] Inger R, Clark P (1943) Partition of the genus *Coluber*. Copeia 3: 141–145.

[12] Nagy Z, Lawson R (2004) Molecular systematics of racers, whipsnakes and relatives (Reptilia: Colubridae) using mitochondrial and nuclear markers. J Zool Syst Evol Res 42: 223–233.

[13] Ortenburger AI (1928) The whip snakes and racers. Genera *Masticophis* and Coluber. Mem Univ Michigan Museum Zool xviii: + 247 pp., 64 figs., 35 pls.

[14] Schätti B, Monsch P (2004) Systematics and phylogenetic relationships of Whip snakes (*Hierophis* Fitzinger) and *Zamenis andreana* Werner, 1917 (Reptilia: Squamata: Colubrinae). Rev suisse Zool 111: 239–256.

[15] Schätti B, Trape J (2008) *Bamanophis*, a new genus for the West African colubrid Periops dorri Lataste, 1888 (Reptilia: Squamata: Colubrinae). Rev suisse Zool 115: 595–615.

[16] Schätti B, Utiger U (2001) *Hemerophis*, a new genus *for Zamenis socotrae* Gunther, and a contribution to the phylogeny of Old World racers, whip snakes, and related genera. Rev Suisse Zool 108: 919–948.

[17] Schätti B, Tillack F, Kucharzewski C (2014) *Platyceps rhodorachis* (JAN, 1863)-a study of the racer genus Platyceps BLYTH, 1860 east of the Tigris (Reptilia: Squamata: Colubridae). Vertebr Zool 64: 297–405.

[18] Vyas R, Patel S (2007) New distributional records of the endemic snake *Coronella brachyura* (Günther 1866) (Serpentes, Colubridae, Colubrinae) from Gujarat State, India. Sauria 29: 47–50.

[19] Whitaker R, Captain A (2004) Snakes of India The field guide. Chennai: Draco Books. 481 p.

[20] Leary S, Underwood W, Anthony R, Cartner S (2013) AVMA guidelines for the euthanasia of animals: 2013 edition: 98.

[21] Ranwez V, Harispe S, Delsuc F, Douzery E (2011) MACSE: Multiple alignment of coding sequences accounting for frameshifts and stop codons. PLoS One 6: e22594 10.1371/journal.pone.0022594 21949676 PMC3174933

[22] Kearse M, Moir R, Wilson A (2012) Geneious Basic: an integrated and extendable desktop software platform for the organization and analysis of sequence data. Bioinformatics 28: 1647–1649. 10.1093/bioinformatics/bts199 22543367 PMC3371832

[23] Stamatakis A (2014) RAxML version 8: a tool for phylogenetic analysis and post-analysis of large phylogenies. Bioinformatics 30: 1312–1313. 10.1093/bioinformatics/btu033 24451623 PMC3998144

[24] Drummond A, Rambaut A (2007) BEAST: Bayesian evolutionary analysis by sampling trees. BMC Evol Biol 7: 214 17996036 10.1186/1471-2148-7-214 PMC2247476

[25] Yu Y, Harris A, Blair C, He X (2015) RASP (Reconstruct Ancestral State in Phylogenies): a tool for historical biogeography. Mol Phylogenet Evol 87: 46–49. 10.1016/j.ympev.2015.03.008 25819445

[26] Holt BG, Lessard JP, Borregaard MK, Fritz SA, Araújo MB, Dimitrov D, et al An update of Wallace’s zoogeographic regions of the world. Science 2013; 339: 74–78. 10.1126/science.1228282 23258408

[27] Dowling H (1951) A proposed standard system of counting ventrals in snakes. Br J Herpetol 11: 97–99.

[28] Anonymous (2014) International Code of Zoological Nomenclature. Int Comm Zool Nomencl. Available: http://iczn.org/iczn/index.jsp.

[29] Champion H, Seth S (2005) A revised survey of the forest types of India Dehradun: Natraj Publishers. 403 p.

[30] Rodgers W, Panwar S (1988) Biogeographical classification of India. New For Dehra Dun, India.

[31] Pyron R, Burbrink F, Wiens J (2013) A phylogeny and revised classification of Squamata, including 4161 species of lizards and snakes. BMC Evol Biol 13: 93 10.1186/1471-2148-13-93 23627680 PMC3682911

[32] Briggs J (2003) The biogeographic and tectonic history of India. J Biogeogr 30: 381–388.

[33] Agarwal I, Iyengar A (2013) Further Records of the Sindh Awl-Headed Snake, *Lytorhynchus paradoxus* (Günther; 1875), from India with Notes on Its Habitat and Natural History. Russ J Herpetol 20: 165–170.

[34] Shackleton N, Kennett J (1975) Paleotemperature history of the Cenozoic and the initiation of Antarctic glaciation: oxygen and carbon isotope analyses in DSDP Sites 277, 279, and 281. Initial reports Deep sea Drill Proj 29: 743–755.

[35] Miller K, Fairbanks R, Mountain G (1987) Tertiary oxygen isotope synthesis, sea level history, and continental margin erosion. Paleoceanography 2: 1–19.

[36] Karanth P (2015) An island called India: phylogenetic patterns across multiple taxonomic groups reveal endemic radiations. Curr Sci 108: 1847–1851.

[37] Losos JB, Ricklefs RE (2009) Adaptation and diversification on islands. Nature 457: 830–836. 10.1038/nature07893 19212401

[38] Queiroz A De (2005) The resurrection of oceanic dispersal in historical biogeography. Trends Ecol Evol 20: 68–73. 16701345 10.1016/j.tree.2004.11.006

[39] Noonan B, Chippindale P (2006) Dispersal and vicariance: the complex evolutionary history of boid snakes. Mol Phylogenet Evol 40: 347–358. 16624591 10.1016/j.ympev.2006.03.010

[40] Vidal N, Marin J, Morini M (2010) Blindsnake evolutionary tree reveals long history on Gondwana. Biol Lett 6: 558–561. 10.1098/rsbl.2010.0220 20356885 PMC2936224

[41] Agarwal I, Bauer AM, Jackman TR, Karanth KP (2014) Insights into Himalayan biogeography from geckos: A molecular phylogeny of *Cyrtodactylus* (Squamata: Gekkonidae). Mol Phylogenet Evol 80: 145–155. 10.1016/j.ympev.2014.07.018 25108260

[42] Gower DJ, Winkler JD (2007) Taxonomy of Indian snake *Xylophis* Beddome (Serpentes: Caenophidia), with description of a new species. Hamadryad 31: 137–151.

[43] Mirza Z, Sanap R (2014) A new cryptic species of gecko of the genus *Hemidactylus* Oken, 1817 (Reptilia: Gekkonidae) from Southern India. Taprobanica 6: 12–20.

[44] Mirza Z, Pal S, Bhosale H, Sanap R (2014) A new species of gecko of the genus *Cnemaspis* Strauch, 1887 from the Western Ghats, India. Zootaxa 3815: 494–506. 10.11646/zootaxa.3815.4.2 24943630

[45] Smith E, Ogale H, Deepak V, Giri V (2012) A new species of coralsnake of the genus *Calliophis* (Squamata: Elapidae) from the west coast of peninsular India. Zootaxa 3437: 51–68.

[46] Vogel G, Rooijen J Van (2011) A new species of *Dendrelaphis* (Serpentes: Colubridae) from the Western Ghats-India. Taprobanica 3: 77–85.

[47] Vogel G, Van Rooijen J (2011) Contributions to the review of *Dendrelaphis pictus* (Gmelin, 1789) complex (Serpentes: Colubridae)-3. The Indian forms, with the description of a new species from Western Ghats. J Herpetol 45: 100–110.

[48] Giri V, Bauer A, Vyas R, Patil S (2009) New species of rock-dwelling *Hemidactylus* (Squamata: Gekkonidae) from Gujarat, India. J Herpetol 43: 385–393.

[49] Vyas R, Giri V, Bauer A (2006) First record of *Hemidactylus persicus* Anderson, 1872 (Squamata: Sauria: Gekkonidae) from the Republic of India, with notes on its distribution. Hamadryad 30:209–211.

[50] Abraham RK, Pyron RA, Ansil BR, Zachariah A, Zachariah A (2013) Two novel genera and one new species of treefrog (Anura: Rhacophoridae) highlight cryptic diversity in the Western Ghats of India. Zootaxa 3640: 177–199. 10.11646/zootaxa.3640.2.3 26000411

[51] Bauer A, Jackman T, Greenbaum E (2010) South Asia supports a major endemic radiation of *Hemidactylus* geckos. Mol Phylogenet Evol 57: 343–352. 10.1016/j.ympev.2010.06.014 20601010

[52] Biju SD, Bossuyt F (2003) New frog family from India reveals an ancient biogeographical link with the Seychelles. Nature 425: 711–7104. 14562102 10.1038/nature02019

[53] Mirza Z A, Sanap RV, Raju D, Gawai A, Ghadekar P (2014) A new species of lizard of the genus *Eublepharis* (Squamata: Eublepharidae) from India. Phyllomedusa 13: 75–90.

