# Correction: A New Miocene-Divergent Lineage of Old World Racer Snake from India

**DOI:** 10.1371/journal.pone.0154301

**Published:** 2016-04-20

**Authors:** Zeeshan A. Mirza, Raju Vyas, Harshil Patel, Jaydeep Maheta, Rajesh V. Sanap

There is an error throughout the manuscript. The spelling for the new genus and species is incorrect. The correct spelling should be: *Wallaceophis gujaratensis*.
