# Supplementary figures and images for: A New Miocene-Divergent Lineage of Old World Racer Snake from India
Source: PLoS One. 2016 Mar 2;11(3):e0148380. doi: 10.1371/journal.pone.0148380 (PMC4774991; doi:10.1371/journal.pone.0148380)

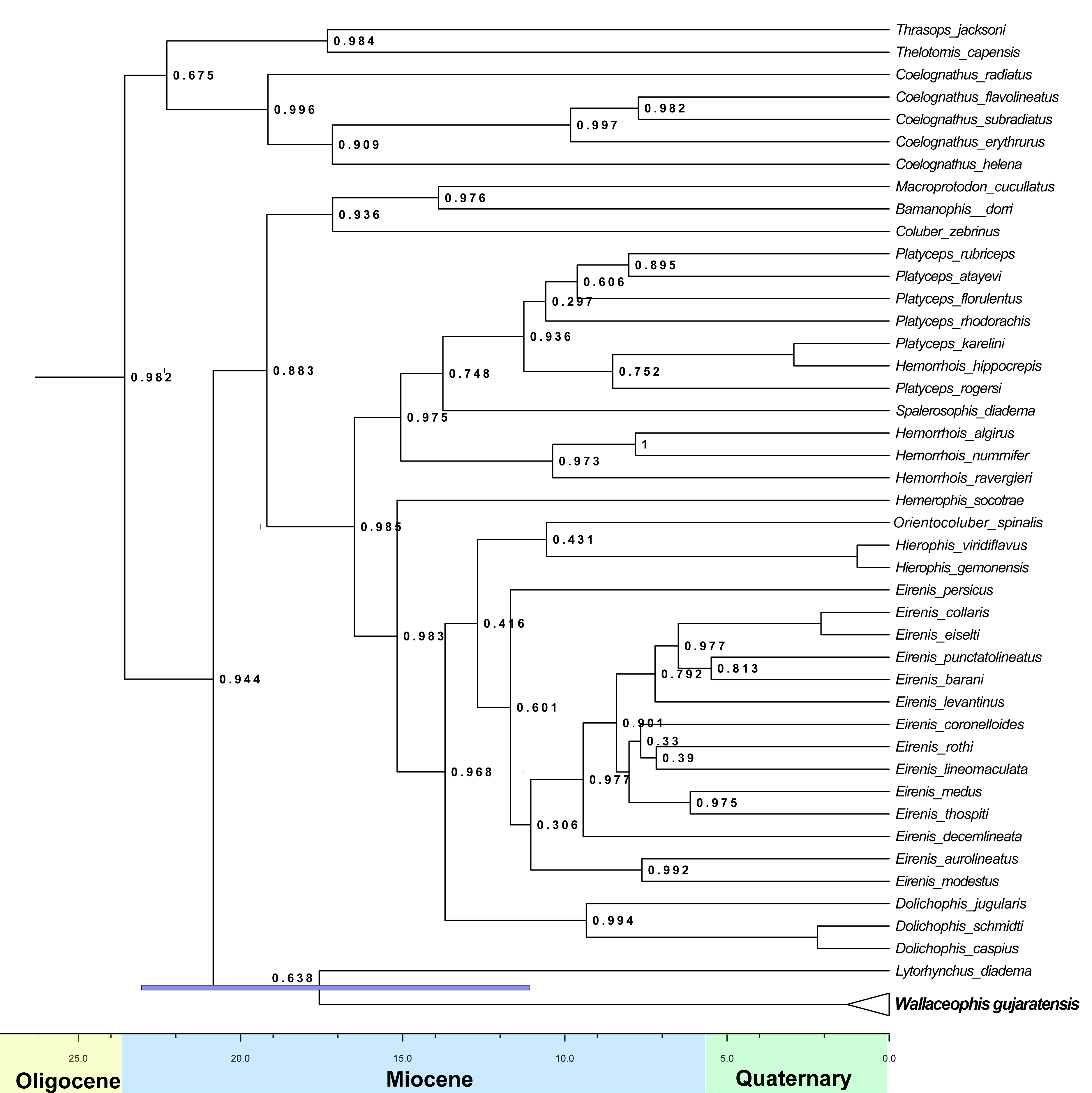

Supplement: S1 Fig — Numbers at node indicate Bayesian posterior probabilities and blue bar indicates 95% HPD. (TIF) [file pone.0148380.s001.tif]
