# Supplementary material for: A New Miocene-Divergent Lineage of Old World Racer Snake from India
Source: PLoS One. 2016 Mar 2;11(3):e0148380. doi: 10.1371/journal.pone.0148380 (PMC4774991; doi:10.1371/journal.pone.0148380)
Supplement: S1 Table — (DOCX) [file pone.0148380.s002.docx]

| Species | cmos | cytb | 16s | ND4 | 12s |
| --- | --- | --- | --- | --- | --- |
| *Eirenis aurolineatus* | AY376807 | AY376749 | AY376778 | AY487070 | - |
| *Eirenis barani* | AY376822 | AY376764 | AY376793 | - | - |
| *Eirenis collaris* | AY376824 | AY376766 | AY376795 | - | - |
| *Eirenis coronelloides* | AY376816 | AY376758 | AY376787 | - | - |
| *Eirenis decemlineatus* | AY376818 | AY376760 | AY376789 | - | - |
| *Eirenis eiselti* | AY376805 | AY376747 | AY376776 | AY487069 | - |
| *Eirenis levantinus* | AY376823 | AY376765 | AY376794 | AY487071 | - |
| *Eirenis lineomaculatus* | AY376820 | AY376762 | AY376791 | - | - |
| *Eirenis medus* | AY376825 | AY376767 | AY376796 | - | AY647226 |
| *Eirenis modestus* | AY486957 | AY486933 | AY039143 | AY487072 | AY376792 |
| *Eirenis punctatolineatus* | AY376813 | AY376755 | AY376784 | AY487073 | AY647227 |
| *Eirenis rothii* | AY376817 | AY376759 | AY376788 | - | - |
| *Eirenis thospitis* | AY376819 | AY376761 | AY376790 | - | - |
| *Hemerophis socotrae* | AY188003 | AY188042 | AY039140 | AY487055 | AY188083 |
| *Hemorrhois algirus* | AY486935 | AY486911 | AY643349 | AY487037 | - |
| *Hemorrhois hippocrepis* | AY486940 | DQ451987 | AY039158 | AY487045 | AY643350 |
| *Hemorrhois nummifer* | AY376800 | AY039201 | AY376771 | AY487049 | - |
| *Hemorrhois ravergieri* | AY486944 | AY486920 | - | AY487050 | - |
| *Heterodon simus* | AF471142 | AF217840 | - | DQ902310 | - |
| *Hierophis gemonensis* | AY376799 | AY039183 | - | AY487044 | - |
| *Orientocoluber spinalis* | AY486948 | AY486924 | AY376773 | AY487056 | - |
| *Hierophis viridiflavus* | AY486949 | AY486925 | AY039142 | AY487057 | AY376774 |
| *Lytorhynchus diadema* | AY187986 | AY188025 | AY643309 |  | AY188064 |
| *Platyceps collaris* | AY486946 | AY486922 | - | AY487053 | AY039157 |
| *Platyceps florulentus* | AY486939 | AY486915 | - | AY487043 | AY039161 |
| *Platyceps karelini* | AY486942 | AY486918 | - | AY487047 | AY647232 |
| *Platyceps najadum* | AY486936 | AY486912 | AY188082 | AY487038 | AY039141 |
| *Platyceps rhodorachis* | AY486945 | AY486921 | - | AY487051 | AY039151 |
| *Platyceps rogersi* | AY188002 | AY188041 | AY188082 | AY487052 | AY188082 |
| *Spalerosophis diadema* | AF471155 | AF471049 | HQ658432 | AY487059 | HQ658450 |
| *Bamanophis dorri* | AY188001 | AY188040 | - | AY487042 | AY188081 |
| *Coluber zebrinus* | AY188004 | AY188043 | - | AY487058 | AY188084 |
| *Pseudocyclophis persicus* | AY376815 | AY376757 | - | - | AY376786 |
| *Dolichophis caspius* | AY376797 | AY039173 | AY039135 | AY487039 | AY039135 |
| *Dolichophis jugularis* | AY486941 | AY486917 | - | AY487046 | AY039152 |
| *Dolichophis schmidti* | AY486947 | AY486923 | - | AY487054 | AY039159 |
| *Macroprotodon cucullatus* | AY187987 | AY188026 | - | AY487064 | AY188065 |
| *Coelognathus erythrurus* | DQ902067 | DQ902108 | - | DQ902288 | AY122665 |
| *Coelognathus flavolineatus* | DQ902090 | DQ902128 | - | DQ902308 | AY122666 |
| *Coelognathus helena* | DQ902071 | DQ902112 | - | DQ902292 | AY122675 |
| *Coelognathus radiata* | DQ902079 | DQ902121 | - | DQ902317 | AY122677 |
| *Coelognathus subradiata* | DQ902084 | DQ902126 | - | DQ902304 | AY122673 |
| *Thrasops jacksonii* | DQ112084 | AF471044 | - | - | - |
| *Thelotornis capensis* | AF471109 | AF471042 | - | - | - |

Appendix A. Genbank accession numbers of sampled taxa for analysis.
